# The effect of general anaesthetics on brain lactate release

**DOI:** 10.1016/j.ejphar.2020.173188

**Published:** 2020-08-15

**Authors:** Anna Hadjihambi, Anastassios Karagiannis, Shefeeq M. Theparambil, Gareth L. Ackland, Alexander V. Gourine

**Affiliations:** a Centre for Cardiovascular and Metabolic Neuroscience, Neuroscience, Physiology and Pharmacology, University College London, London, WC1E 6BT, UK; b Department of Biomedical Sciences, University of Lausanne, Lausanne, 1005, Switzerland; c Translational Medicine & Therapeutics, William Harvey Research Institute, Queen Mary University of London, London, EC1M 6BQ, UK

**Keywords:** Anaesthesia, Brain energy metabolism, GABAergic mechanisms, Lactate, Lactate shuttle, Sleep

## Abstract

The effects of anaesthetic agents on brain energy metabolism may explain their shared neurophysiological actions but remain poorly understood. The brain lactate shuttle hypothesis proposes that lactate, provided by astrocytes, is an important neuronal energy substrate. Here we tested the hypothesis that anaesthetic agents impair the brain lactate shuttle by interfering with astrocytic glycolysis. Lactate biosensors were used to record changes in lactate release by adult rat brainstem and cortical slices in response to thiopental, propofol and etomidate. Changes in cytosolic nicotinamide adenine dinucleotide reduced (NADH) and oxidized (NAD^+^) ratio as a measure of glycolytic rate were recorded in cultured astrocytes. It was found that in brainstem slices thiopental, propofol and etomidate reduced lactate release by 7.4 ± 3.6% (P < 0.001), 9.7 ± 6.6% (P < 0.001) and 8.0 ± 7.8% (P = 0.04), respectively. In cortical slices, thiopental reduced lactate release by 8.2 ± 5.6% (P = 0.002) and propofol by 6.0 ± 4.5% (P = 0.009). Lactate release in cortical slices measured during the light phase (period of sleep/low activity) was ~25% lower than that measured during the dark phase (period of wakefulness) (326 ± 83 μM *vs* 430 ± 118 μM, n = 10; P = 0.04). Thiopental and etomidate induced proportionally similar decreases in cytosolic [NADH]:[NAD^+^] ratio in astrocytes, indicative of a reduction in glycolytic rate. These data suggest that anaesthetic agents inhibit astrocytic glycolysis and reduce the level of extracellular lactate in the brain. Similar reductions in brain lactate release occur during natural state of sleep, suggesting that general anaesthesia may recapitulate some of the effects of sleep on brain energy metabolism.

## 1 Introduction

Anaesthetic agents induce a reversible state of unconsciousness by altering the neuronal activity through enhancing inhibitory, and/or inhibiting excitatory synaptic interactions (Garcia et al., 2010). Many injectable anaesthetics, including propofol (fast-acting nonbarbiturate), etomidate (nonbarbiturate imidazolic derivative), and thiopental (barbiturate) share a common mechanism of action by enhancing the efficacy of signalling via the inhibitory gamma-aminobutyric acid A (GABA_A_) receptors (Canbek et al., 2015). Anaesthesia and sleep may share some common neurophysiological mechanisms (Vacas et al., 2013), including suppression of arousal via enhanced activity of GABAergic pathways (Lu et al., 2008). For example, in brain regions that promote wakefulness, such as dorsal raphé, hypothalamic tuberomammillary nucleus, medial preoptic area and others, facilitated GABAergic inhibition has been shown to promote sleep, – an effect similar to that of anaesthetic agents that target GABA_A_ receptor-mediated mechanisms (Watson et al., 2010).

CNS neurons require a constant energy supply that matches their high metabolic demands. According to the astrocyte-to-neuron lactate shuttle hypothesis, lactate is a major neuronal energy substrate (Pellerin et al., 1998). Lactate is supplied to neurons by neighbouring astrocytes following glycolytic breakdown of glucose and release via monocarboxylate transporters (MCTs) (Perez-Escuredo et al., 2016) and/or certain membrane channels (Sotelo-Hitschfeld et al., 2015; Karagiannis et al., 2016; Hadjihambi et al., 2017). There is evidence that reduced glucose metabolism in astrocytes delays emergence from general anaesthesia (Ramadasan-Nair et al., 2019). However, the effects of general anaesthesia on brain energy metabolism are poorly understood. Changes in cerebral blood flow and cerebral metabolic rate of oxygen have been reported with the use of propofol (Kaisti et al., 2003), etomidate (Cold et al., 1985) and thiopental (Wechsler et al., 1951). Limited data exists on the effects of anaesthetic agents on brain lactate metabolism. A microdialysis study (Horn and Klein, 2010) reported an increase in lactate concentration in the mouse brain upon exposure to isoflurane. By contrast, results of another study that used ^13^C-magnetic resonance spectroscopy suggested that cortical rates of oxidative metabolism in both glia and neurons are reduced under thiopental anaesthesia (Sonnay et al., 2017). The authors of the latter report proposed that lactate is used as a substitute metabolic substrate upon exposure to this anaesthetic agent (Sonnay et al., 2017).

This study tested the hypothesis that anaesthetic agents exert their effects, at least in part, through the inhibition of the astrocyte-to-neuron lactate shuttle. The data obtained show that propofol, etomidate and thiopental reduce the release of lactate in the brain. The effects are similar to that of a naturally occurring state of sleep, suggesting that general anaesthesia may recapitulate some of the effects of sleep on brain energy metabolism.

## 2 Materials & methods

All experiments were performed in accordance with the European Commission Directive 2010/63 (European Convention for the Protection of Vertebrate Animals used for Experimental and Other Scientific Purposes) and the UK Home Office (Scientific Procedures) Act (1986) with project approval by the Institutional Animal Welfare and Ethical Review Committee of the University College London (Ref PECE77103). ARRIVE guidelines were adhered to.

### 2.1 Slice preparation

Young adult male Sprague-Dawley rats (~200 g, n = 100), supplied by the University College London animal facility were group housed (3 per cage) in individually ventilated cages kept on a 12 h light-dark cycle (light 07:00-19:00), with *ad libitum* access to standard rat chow and water. Environmental enrichment was provided in the form of chew sticks and cardboard tubes. In the laboratory, the animals were terminally anesthetized with halothane inhalation overdose and the brains were removed and placed in ice cold artificial cerebrospinal fluid (aCSF) containing 124 mM NaCl, 26 mM NaHCO_3_, 3 mM KCl, 2 mM CaCl_2_, 1.25 mM NaH_2_PO_4_, 1 mM MgSO_4_, 10 mM Glucose saturated with 95% O_2_ and 5% CO_2_ (pH 7.4) with an addition of 9 mM Mg^2+^. Coronal cortical (cut at the level of the somatosensory cortex) and brainstem slices (thickness 300 μm) were prepared using a vibratome and then incubated at room temperature for 1 h in a standard aCSF saturated with 95% O_2_ and 5% CO_2_.

### 2.2 Recording lactate release

**Fig. 1:**
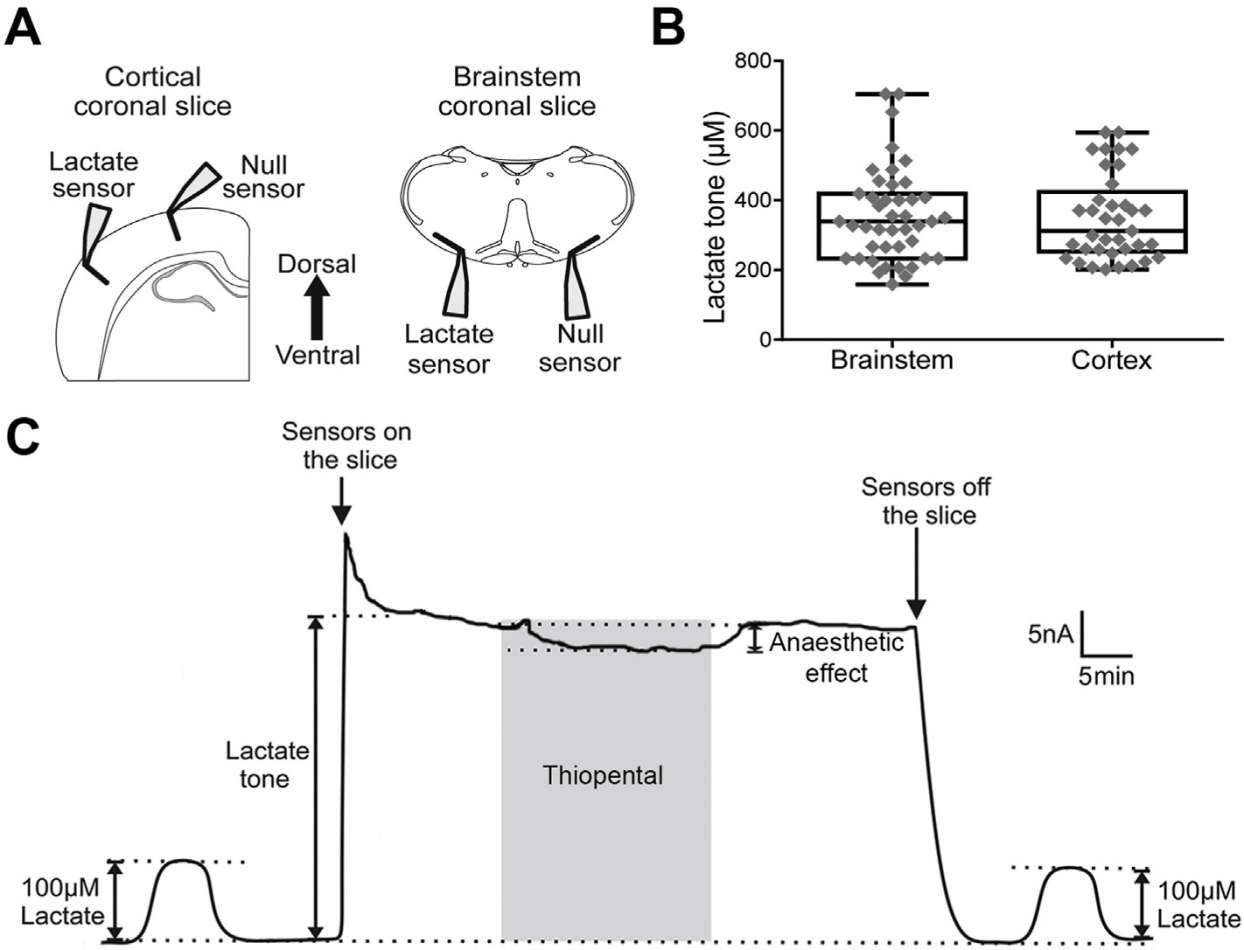
**Biosensor recordings of lactate release in cortical and brainstem slices**. **A)** Schematic drawings of the dual recording configuration of lactate and null (control) biosensors placed on the surfaces of cortical and brainstem slices. **B)** Summary data illustrating tonic lactate release (lactate tone) recorded in 43 brainstem and 37 cortical slices. **C)** Representative example of changes in the net lactate biosensor current (difference in current between the lactate and null sensors) during calibration (100 μM lactate), after biosensor placement in the direct contact with the surface of the cortical slice (recording tonic lactate release) and in response to application of thiopental (10 μM).

Recordings were made from the brain slices placed on an elevated grid in a flow chamber (flow 3 ml/min) at 35°C. Release of lactate was recoded in real-time using amperometric enzymatic lactate biosensors (Sarissa Biomedical) placed in a direct contact with the surface of the slice (Fig. 1A). The operation of the lactate biosensor is based on the enzymatic activity of lactate oxidase which, in the presence of oxygen, converts lactate to pyruvate and H_2_O_2_. The latter is detected electrochemically. A dual recording configuration of a null sensor (lacking lactate oxidase) placed on the surface of the slice along the lactate biosensor was used, as described in detail previously (Gourine et al., 2005, 2008; Karagiannis et al., 2016; Hadjihambi et al., 2017) (Fig. 1A). The null sensor was used to control for the potential release of non-specific electroactive interferents that could confound the measurements. Null sensor currents were subtracted from the lactate biosensor currents. Biosensors were calibrated directly in the recording chamber immediately before and after every recording by application of 100 μM lactate (Fig. 1C). The initial and final calibrations were used to determine the sensor sensitivity changes during the experiment. The sensor currents were converted to lactate concentrations according to this calibration (Fig. 1C).

To determine the differences in brain lactate release between sleep and wakefulness, the rats were killed, and cortical slices were prepared either during the dark phase (wakefulness) or light phase (sleep/low activity) of the 24 h light-dark cycle. The experiments were performed between 12:00-16:00 for the light phase (lights on 07:00-19:00) or 00:00-06:00 for the dark phase (lights off 19:00-07:00). All the experiments involving testing the effects of the drugs on tonic lactate release were performed between 12:00-16:00.

### 2.3 Primary astrocyte cultures

Primary astrocyte cultures were prepared from the brains of rat pups (p2-p5 of either sex) as described previously (Theparambil et al., 2014; Turovsky et al., 2016; Sheikhbahaei et al., 2018). After isolation, the cells were plated on Poly-L-Lysine coated glass coverslips (15 mm) and maintained in Dulbecco's Modified Eagle's Medium (DMEM) containing 5% fetal calf serum in a humidified atmosphere of 5% CO_2_ and 95% air at 37°C. The culture medium was replaced after 24 h of cell plating and subsequently every 3 days.

### 2.4 Recording changes in cytosolic nicotinamide adenine dinucleotide (reduced, NADH; oxidized, NAD^+^) [NADH]:[NAD^+^] ratio is astrocytes

After 7 days of culturing, astrocytes were transduced to express a genetically-encoded [NADH]/[NAD^+^] sensor *Peredox* using an adenoviral vector AVV-CMV-Peredox (Hung et al., 2011). The cells were used for imaging experiments after 3 days of the transfection. Recordings were made in aCSF in a flow chamber (flow 1 ml/min) at 35°C. An Olympus IX71×inverted microscope, 20x oil immersion objective lens, cooled CCD camera (Clara, Andor) and Andor iQ3 software were used for data acquisition. The green T-Sapphire and red mCherry proteins were alternatively excited using 405/10 nm and 575/10 nm wavelengths at a frequency of 0.2 Hz. The fluorescence emission was recorded at 535/30 nm and 632/60 nm.

### 2.5 Drugs

Once the biosensor recordings of tonic lactate release or *Peredox* fluorescence emission had stabilized (~15 min), anaesthetic agents were bath applied and the changes in the release of lactate or cytosolic [NADH]:[NAD^+^] ratio were recorded. Muscimol was applied at a concentration of 100 μM (Cheung et al., 2009). Propofol, thiopental and etomidate were applied at 10 μM. Propofol was obtained from Aspen Pharma. All other drugs were obtained from Sigma, diluted in the solvent suggested by the supplier and added to the perfusate at the indicated concentration.

### 2.6 Statistical analysis

Lactate biosensor recordings were processed using Power 1401 interface and analysed using *Spike 2* software (Cambridge Electronic Design) and Prism software (GraphPad 7.0). Based on our data obtained previously with the use of amperometric biosensors, a sample size of at least 10 was calculated to give 90% power (alpha = 0.05) (Gpower 3 v3.1.9.2). Data are reported as individual data points and box and whisker plots. Datasets were tested for normality and were compared using paired samples Wilcoxon Signed Ranks Test or Student's paired or unpaired *t-*test, as appropriate. The results of the imaging experiments are reported as bar charts and the group data were compared using Student's paired *t*-test. Values in the text are expressed as means ± S.D. Differences between the experimental groups with P values of less than 0.05 were considered to be significant. All reported n numbers indicate the numbers of animals.

## 3 Results

**Fig. 2:**
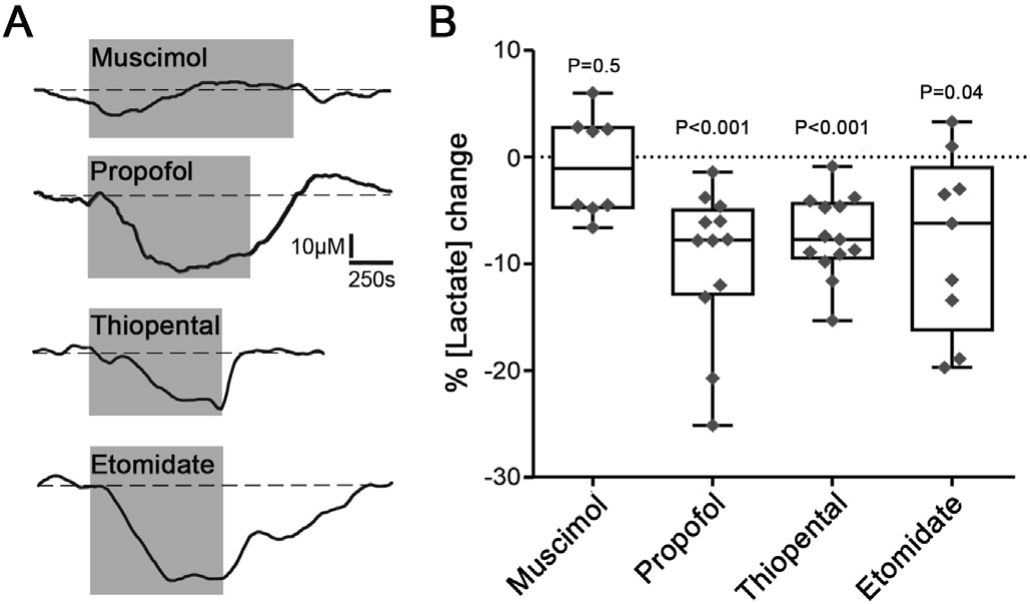
**Changes in lactate release recorded in brainstem slices in response to the anaesthetic agents**. **A)** Representative recordings of lactate biosensor currents showing changes in tonic lactate release in response to test drugs. Dashed lines show the extrapolation of lactate biosensor current prior to the application of the drug. **B)** Summary data illustrating the magnitude of changes in tonic lactate release in response to application of muscimol (100 μM), propofol (10 μM), thiopental (10 μM) or etomidate (10 μM).

**Fig. 3:**
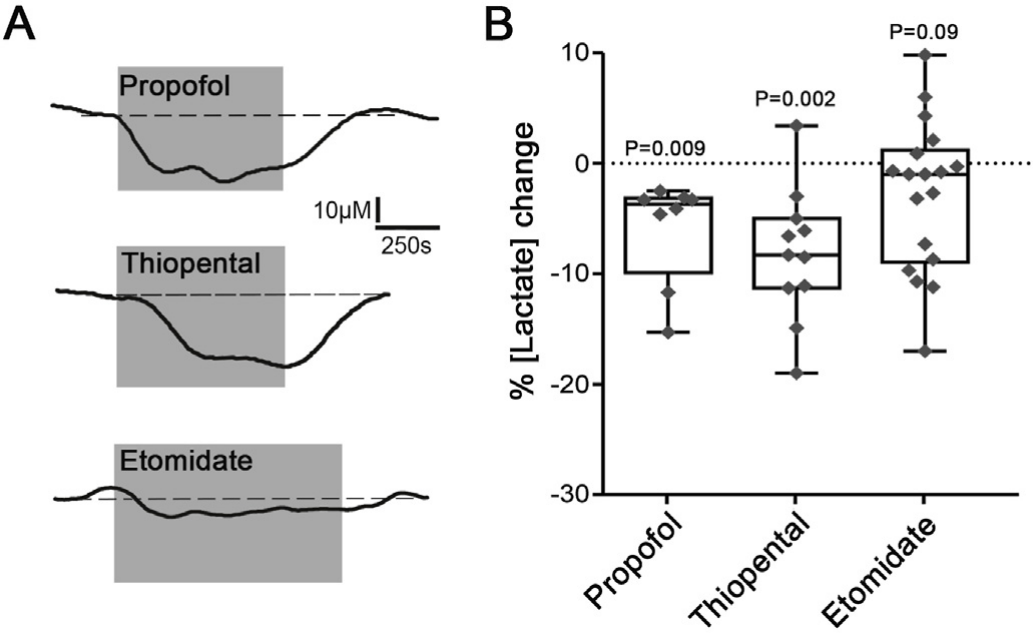
**Changes in lactate release recorded in cortical slices in response to the anaesthetic agents**. **A)** Representative recordings of lactate biosensor currents showing changes in tonic lactate release in response to test drugs. Dashed lines show the extrapolation of lactate biosensor current prior to the application of the drug. **B)** Summary data illustrating the magnitude of changes in tonic lactate release in response to application of propofol (10 μM), thiopental (10 μM) or etomidate (10 μM).

The lactate biosensors were placed either across all the layers of the cortex or close to the ventral surface of the brainstem (Fig. 1A). Significant tonic lactate release (lactate tone) was detected by the biosensors at the surface of the brainstem (357 ± 131 μM; n = 43, P < 0.001) and cortical (350 ± 122 μM; n = 37, P < 0.001) slices (Fig. 1B). Once the measurement of tonic lactate release had stabilized, anaesthetic agents were applied and the changes in the release of lactate were recorded (Fig. 1C). In brainstem slices, thiopental (10 μM), propofol (10 μM) and etomidate (10 μM) had similar effects and reduced the release of lactate by 7.4 ± 3.6% (n = 13, P < 0.001), 9.7 ± 6.6% (n = 12, P < 0.001) and 8.0 ± 7.8% (n = 9, P = 0.04), respectively (Fig. 2). In cortical slices, thiopental and propofol reduced lactate release by 8.2 ± 5.6% (n = 11, P = 0.002) and 6.0 ± 4.5% (n = 8, P = 0.009), respectively (Fig. 3). Etomidate in the concentration used (10 μM) had no effect on tonic lactate release recorded in the cortical slices (2.8 ± 6.8%; n = 18, P = 0.09) (Fig. 3). Muscimol, a potent GABA_A_ agonist, applied in concentration 100 μM had no effect on brain lactate release (0.9 ± 4.5%; n = 8, P = 0.5) (Fig. 2).

**Fig. 4:**
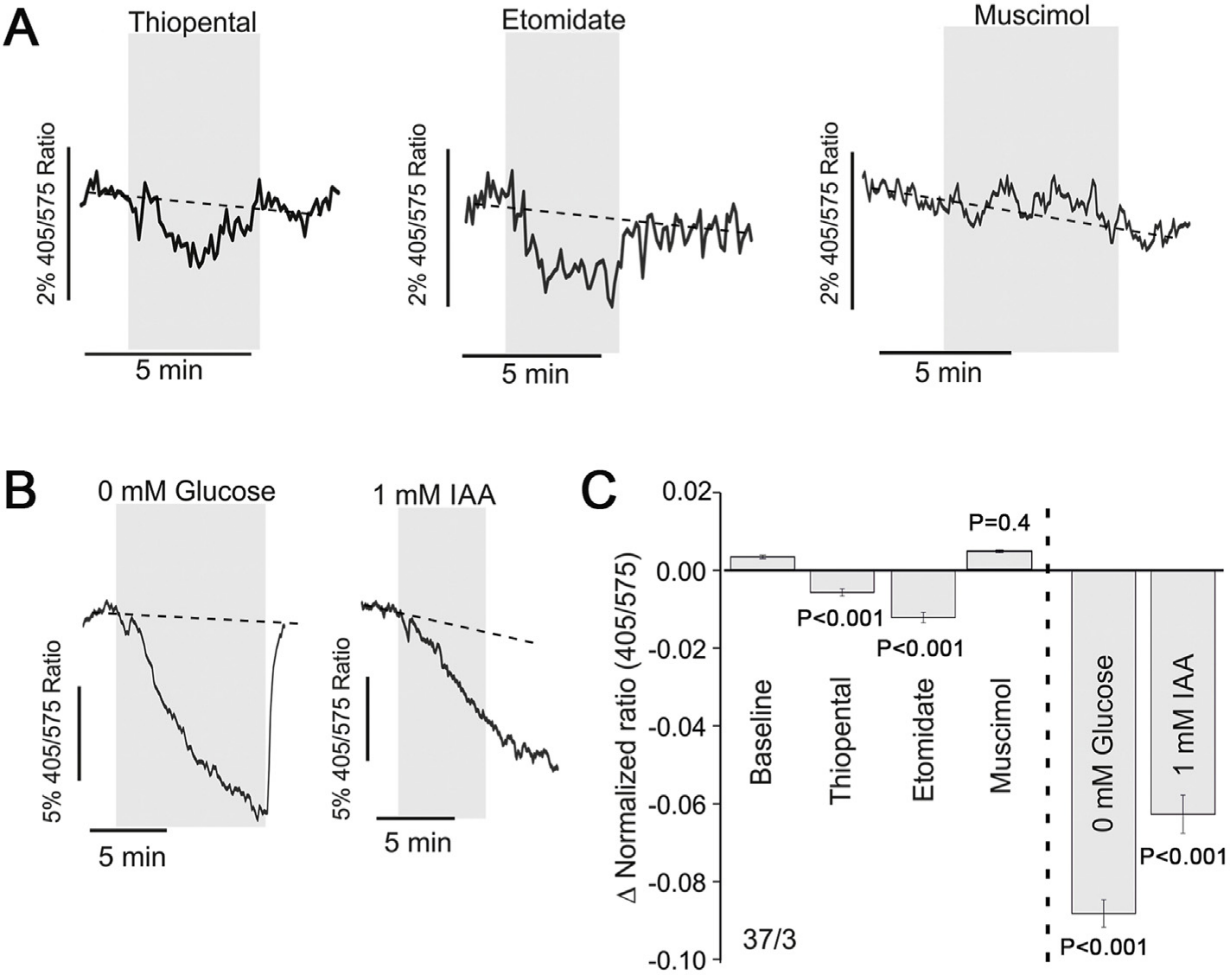
**Changes in cytosolic [NADH]:[NAD**^**+**^**] ratio in cultured astrocytes in response to the anaesthetic agents**. **A)** Representative recordings of *Peredox* fluorescence changes illustrating the effect of thiopental (10 μM), etomidate (10 μM) and muscimol (100 μM) on cytosolic [NADH]:[NAD^+^] ratio in cultured astrocytes. Dashed lines show the extrapolation of the *Peredox* fluorescence prior to the application of the test drug. **B)** Representative recordings of *Peredox* fluorescence changes illustrating the effect of 0 mM glucose and iodoacetic acid (IAA, irreversible inhibitor of glycolysis) on cytosolic [NADH]:[NAD^+^] ratio. Dashed lines show the extrapolation of the baseline *Peredox* fluorescence. **C)** Summary data illustrating the effects of thiopental, etomidate, muscimol, glucose deprivation and IAA on cytosolic [NADH]:[NAD^+^] ratio in cultured astrocytes.

There is significant evidence that astrocytes (as highly glycolytic cells) are the net produces of lactate that is transported to neurons in accord with the concentration gradient of this metabolite (Machler et al., 2016; Barros and Weber, 2018). High cytosolic [NADH]:[NAD^+^] ratio in astrocytes is primarily conferred by glycolysis (Hung et al., 2011; Kohler et al., 2018), therefore, it can be used as a measure of glycolytic rate (Hung et al., 2011). It was found that both thiopental (P < 0.001) and etomidate (P < 0.001) reversibly decreased the cytosolic [NADH]:[NAD^+^] ratio in astrocytes (Fig 4A, C), indicating that these agents reduce the glycolytic rate. The effect of propofol was not tested because the optical properties of propofol solution interfered with the measurements of changes in *Peredox* fluorescence. Muscimol had no effect on cytosolic [NADH]:[NAD^+^] ratio in astrocytes (P = 0.3, Fig. 4A, C). Changes in [NADH]:[NAD^+^] ratio in astrocytes induced by anaesthetic agents were compared to that induced by glucose deprivation (0 mM glucose in aCSF) or irreversible inhibition of glycolysis with iodoacetic acid (IAA, 1 mM). Both glucose deprivation (P < 0.001) and IAA (P < 0.001) caused marked reductions in cytosolic [NADH]:[NAD^+^] ratio (Fig. 4B, C). The effect of thiopental on [NADH]:[NAD^+^] ratio in astrocytes was calculated to be 7% of that induced by glucose deprivation and 10% of that induced by IAA. The inhibitory effect of etomidate on astrocytic glycolysis was 13% of that induced by glucose deprivation and 19% of that induced by IAA.

**Fig. 5:**
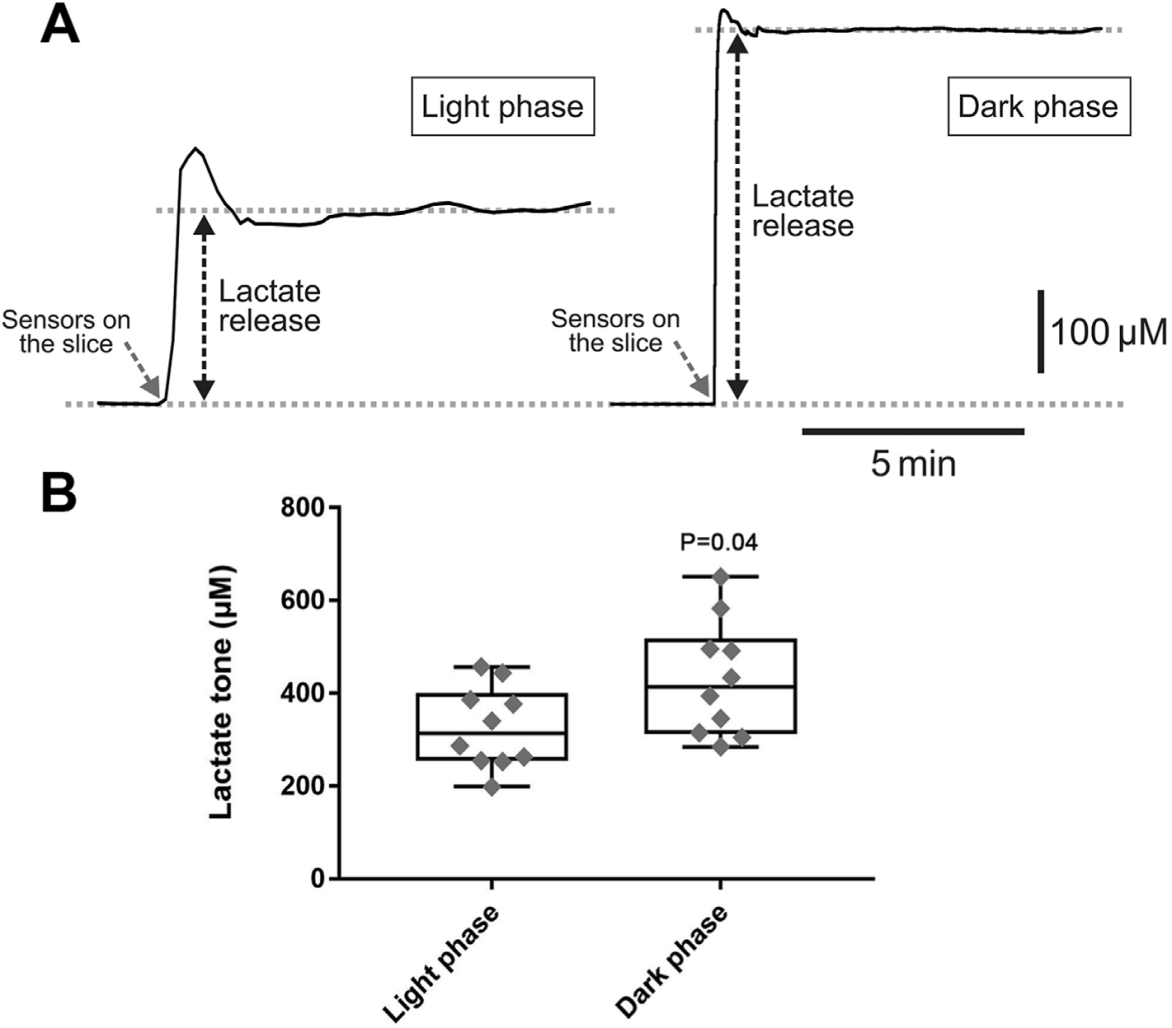
**Lactate release recorded in cortical slices of animals killed during the light and the dark phases of the 24 h cycle**. **A)** Representative recordings and **B)** summary data of net lactate biosensor currents after biosensor placement in the direct contact with the surface of the cortical slices (recording tonic lactate release) prepared from the brains of animals during the light phase (sleep/low activity) and dark phase (wakefulness).

The effects of anaesthetic agents on brain lactate release were next compared to that occurring naturally during the transition from wakefulness to sleep. It was found that tonic lactate release in cortical slices of animals killed during the light phase (period of sleep/low activity) was ~25% lower than that of animals killed during the dark phase (period of wakefulness) (326 ± 83 μM, n = 10 *vs* 430 ± 118 μM, n = 10; P = 0.04) (Fig. 5).

## 4 Discussion

The effects of general anaesthesia on brain energy metabolism remain incompletely understood. Although several studies investigated the effects of anaesthetic agents on brain metabolism (Horn and Klein, 2010; Liu et al., 2016; Sonnay et al., 2017), most of the data appear to be inconclusive or conflicting, likely due to the distinct CNS targets of different anaesthetic agents (Sonner et al., 2003; Forman and Chin, 2008). Moreover, while there is an ongoing debate on whether the state of general anaesthesia resembles natural sleep (Vacas et al., 2013), changes in brain energy metabolism under general anaesthesia and sleep have not been comparatively assessed.

This study tested the hypothesis that anaesthetic agents may act via the impairment of the astrocyte-to-neuron lactate shuttle and, by doing so, reduce supply of energy substrate(s) to the CNS neurons and lower the brain activity. We selected three anaesthetic agents, propofol, thiopental and etomidate, all known to facilitate signalling mediated by GABAergic mechanisms, and investigated the effects of these agents on lactate release by the cerebral cortex and the brainstem. Experiments were conducted in acute brain slices to exclude the systemic effects of the drugs. The chosen concentrations of propofol and etomidate (both at 10 μM) were considered to be clinically relevant based on the results of published studies that investigated the diffusion characteristics and potencies of these drugs (Benkwitz et al., 2007). With 10 μM of etomidate, slice tissue concentration of ≥4 μM is expected to be reached during the application period (Voss et al., 2016), which is within the range (4-12 μM) required to induce a state of deep anaesthesia in experimental animals (De Paepe et al., 1999). Clinical concentrations of thiopental range between 10 and 100 μM (Pryor et al., 2004; Veselis et al., 2004; Papatheodoropoulos et al., 2007). The use of 10 μM thiopental was selected because when this concentration in the brain is reached, the experimental animals lose their righting reflex (Gustafsson et al., 1996) and humans fail to respond to verbal commands (Hung et al., 1992). There is also evidence that thiopental in this concentration prolongs postsynaptic inhibitory currents (Dickinson et al., 2002), and reduces the efficacy of gap junction-mediated signalling (Wentlandt et al., 2006).

The data obtained showed that propofol, thiopental and etomidate reduce the release of lactate in the brain. The application of a potent GABA_A_ receptor agonist muscimol had no effect, suggesting that the decreases in lactate release in response to these anaesthetic agents are not due to the actions of these agents on GABA_A_ receptor-mediated mechanisms.

According to the results of earlier studies using the same lactate biosensors (Karagiannis et al., 2016), lactate tone recorded on the intact (uncut) brainstem surface was similar to that measured by the biosensors placed in a direct contact with the surface of the coronal brain sections. The same study reported that lactate tone detected on the slice surface was markedly and reversibly reduced by application of the lactate dehydrogenase inhibitor oxamate (Karagiannis et al., 2016). These data confirmed the analyte selectivity of the lactate biosensor detection system and indicated that the damaged/dying cells are not the primary source of lactate release in this preparation.

While general anaesthesia is usually described as a “reversible drug-induced coma”, rather than deep sleep (Brown et al., 2010), a study by Thrane et al. (2012) demonstrated in rodents that different anaesthetic agents induce electrocorticogram activity changes closely resembling those occurring during sleep. The data obtained in our study showed that tonic lactate release in cortical slices of animals killed during the light phase (period of sleep/low activity) was lower than that of animals killed during the dark phase (period of wakefulness). Therefore, the effects of anaesthetics on extracellular lactate in the brain resemble changes induced by a natural state of sleep and may suggest the existence of some common underlying mechanisms.

That the brain lactate concentration is higher during wakefulness than during sleep has been reported previously (Shram et al., 2002; Naylor et al., 2012; Lundgaard et al., 2017). Shram and colleagues (Shram et al., 2002) observed a 16% reduction of brain lactate concentration during slow wave sleep in freely behaving rats. Lundgaard et al. (2017) reported a 20-30% reduction in cortical extracellular lactate during transition from the dark phase to light phase in mice, the effect that the authors explained by enhanced lactate clearance via the glymphatic system during sleep. In this study we observed quantitively similar (~25%) reduction of tonic lactate release recorded in cortical slices prepared from the brains of animals during their period of sleep/low activity, the difference that cannot be explained by facilitated clearance via convective flow and fluid exchange.

Measurements of cytosolic [NADH]:[NAD^+^] ratio in astrocytes demonstrated that thiopental and etomidate have a direct effect on these cells and inhibit astrocytic glycolytic rate. These data suggest that anaesthetic agents decrease brain extracellular lactate concentration by interfering with glucose metabolism in astrocytes. However, we cannot exclude the possibility that anaesthetics may also have an effect on the mechanisms of lactate release that involve MCTs (Perez-Escuredo et al., 2016) and/or membrane channels, including connexin and pannexin hemichannels (Sotelo-Hitschfeld et al., 2015; Karagiannis et al., 2016). Indeed, there is evidence (Wentlandt et al., 2006) that some anaesthetic agents inhibit gap junctions and gap junction hemichannels expressed by astrocytes and neurons. We previously reported that hemichannels function as conduits of lactate release (Karagiannis et al., 2016), supporting the hypothesis that anaesthetic agents may also act through inhibition of hemichannel activity. This hypothesis is also supported by the evidence of a significant concentration gradient between astrocytes and neurons that favours astrocyte-to-neuron flow of lactate (Machler et al., 2016).

This study aimed to address the effect of general anaesthesia on brain energy metabolism focusing on astrocytes as the major source of lactate, highlighted in the recent editorial (Perouansky et al., 2019). The strength of this study is that different agents were used and showed similar effects on brain lactate release and astrocytic glycolysis. Our experiments focused on the glycolytic aspect of brain energy metabolism and more specifically on the supply of lactate by astrocytes to support the metabolic needs of active neurons, which compliment recently published works in the field (Ramadasan-Nair et al., 2019). In addition, changes in brain lactate release under anaesthesia and sleep have been comparatively assessed. An additional strength of this study is that the experiments were conducted *in vitro* and the effects of anaesthetic agents on brain tissue lactate release were not confounded by systemic effects or the effects of these drugs on the efficacy of lactate clearance by the brain fluid exchange mechanisms (Lundgaard et al., 2017). The use of slice specific calibrations allowed comparisons of the effects between different experimental conditions.

## 5 Conclusions

In conclusion, the results of this study demonstrate that anaesthetic agents inhibit astrocytic glycolysis and reduce the level of extracellular lactate in the brain. These effects are similar to that of a naturally occurring state of sleep, suggesting that general anaesthesia may recapitulate some of the effects of sleep on brain energy metabolism.

## Funding

This study was supported by 10.13039/100010269The Wellcome Trust (to A.V.G.) and British Oxygen Company research chair grant in anaesthesia from the 10.13039/501100001297Royal College of Anaesthetists (to G.L.A.). A.V.G. is a 10.13039/100010269Wellcome Trust Senior Research Fellow (Ref: 200893).

## CRediT authorship contribution statement

**Anna Hadjihambi:** Validation, Investigation, Writing - original draft, Visualization, Formal analysis. **Anastassios Karagiannis:** Formal analysis, Investigation, Visualization. **Shefeeq M. Theparambil:** Investigation, Visualization, Formal analysis. **Gareth L. Ackland:** Resources, Writing - review & editing. **Alexander V. Gourine:** Conceptualization, Resources, Writing - review & editing, Supervision, Funding acquisition.

## Declaration of competing interest

G.L.A is a member of the editorial advisory board for *Intensive Care* *Medicine Experimental*, is an Editor for the *British Journal of Anaesthesia*, and has undertaken consultancy work for GlaxoSmithKline. There are no other relationships or activities that could appear to have influenced the submitted work.

## References

[bib1] Barros L.F., Weber B. (2018). CrossTalk proposal: an important astrocyte-to-neuron lactate shuttle couples neuronal activity to glucose utilisation in the brain. J. Physiol..

[bib2] Benkwitz C., Liao M., Laster M.J., Sonner J.M., Eger E.I., Pearce R.A. (2007). Determination of the EC50 amnesic concentration of etomidate and its diffusion profile in brain tissue: implications for in vitro studies. Anesthesiology.

[bib3] Brown E.N., Lydic R., Schiff N.D. (2010). General anesthesia, sleep, and coma. N. Engl. J. Med..

[bib4] Canbek O., Ipekcioglu D., Menges O.O., Atagun M.I., Karamustafalioglu N., Cetinkaya O.Z., Ilnem M.C. (2015). Comparison of propofol, etomidate, and thiopental in anesthesia for electroconvulsive therapy: a randomized, double-blind clinical trial. J. ECT.

[bib5] Cheung G., Kann O., Kohsaka S., Faerber K., Kettenmann H. (2009). GABAergic activities enhance macrophage inflammatory protein-1alpha release from microglia (brain macrophages) in postnatal mouse brain. J. Physiol..

[bib6] Cold G.E., Eskesen V., Eriksen H., Amtoft O., Madsen J.B. (1985). CBF and CMRO2 during continuous etomidate infusion supplemented with N2O and fentanyl in patients with supratentorial cerebral tumour. A dose-response study. Acta Anaesthesiol. Scand..

[bib7] De Paepe P., Van Hoey G., Belpaire F.M., Rosseel M.T., Boon P.A., Buylaert W.A. (1999). Relationship between etomidate plasma concentration and EEG effect in the rat. Pharm. Res. (N. Y.).

[bib8] Dickinson R., de Sousa S.L., Lieb W.R., Franks N.P. (2002). Selective synaptic actions of thiopental and its enantiomers. Anesthesiology.

[bib9] Forman S.A., Chin V.A. (2008). General anesthetics and molecular mechanisms of unconsciousness. Int. Anesthesiol. Clin..

[bib10] Garcia P.S., Kolesky S.E., Jenkins A. (2010). General anesthetic actions on GABA(A) receptors. Curr. Neuropharmacol..

[bib11] Gourine A.V., Dale N., Korsak A., Llaudet E., Tian F., Huckstepp R., Spyer K.M. (2008). Release of ATP and glutamate in the nucleus tractus solitarii mediate pulmonary stretch receptor (Breuer-Hering) reflex pathway. J. Physiol..

[bib12] Gourine A.V., Llaudet E., Dale N., Spyer K.M. (2005). ATP is a mediator of chemosensory transduction in the central nervous system. Nature.

[bib13] Gustafsson L.L., Ebling W.F., Osaki E., Stanski D.R. (1996). Quantitation of depth of thiopental anesthesia in the rat. Anesthesiology.

[bib14] Hadjihambi A., De Chiara F., Hosford P.S., Habtetion A., Karagiannis A., Davies N., Gourine A.V., Jalan R. (2017). Ammonia mediates cortical hemichannel dysfunction in rodent models of chronic liver disease. Hepatology.

[bib15] Horn T., Klein J. (2010). Lactate levels in the brain are elevated upon exposure to volatile anesthetics: a microdialysis study. Neurochem. Int..

[bib16] Hung O.R., Varvel J.R., Shafer S.L., Stanski D.R. (1992). Thiopental pharmacodynamics. II. Quantitation of clinical and electroencephalographic depth of anesthesia. Anesthesiology.

[bib17] Hung Y.P., Albeck J.G., Tantama M., Yellen G. (2011). Imaging cytosolic NADH-NAD(+) redox state with a genetically encoded fluorescent biosensor. Cell Metabol..

[bib18] Kaisti K.K., Langsjo J.W., Aalto S., Oikonen V., Sipila H., Teras M., Hinkka S., Metsahonkala L., Scheinin H. (2003). Effects of sevoflurane, propofol, and adjunct nitrous oxide on regional cerebral blood flow, oxygen consumption, and blood volume in humans. Anesthesiology.

[bib19] Karagiannis A., Sylantyev S., Hadjihambi A., Hosford P.S., Kasparov S., Gourine A.V. (2016). Hemichannel-mediated release of lactate. J. Cerebr. Blood Flow Metabol..

[bib20] Kohler S., Winkler U., Sicker M., Hirrlinger J. (2018). NBCe1 mediates the regulation of the NADH/NAD(+) redox state in cortical astrocytes by neuronal signals. Glia.

[bib21] Liu X., Gangoso E., Yi C., Jeanson T., Kandelman S., Mantz J., Giaume C. (2016). General anesthetics have differential inhibitory effects on gap junction channels and hemichannels in astrocytes and neurons. Glia.

[bib22] Lu J., Nelson L.E., Franks N., Maze M., Chamberlin N.L., Saper C.B. (2008). Role of endogenous sleep-wake and analgesic systems in anesthesia. J. Comp. Neurol..

[bib23] Lundgaard I., Lu M.L., Yang E., Peng W., Mestre H., Hitomi E., Deane R., Nedergaard M. (2017). Glymphatic clearance controls state-dependent changes in brain lactate concentration. J. Cerebr. Blood Flow Metabol..

[bib24] Machler P., Wyss M.T., Elsayed M., Stobart J., Gutierrez R., von Faber-Castell A., Kaelin V., Zuend M., San Martin A., Romero-Gomez I., Baeza-Lehnert F., Lengacher S., Schneider B.L., Aebischer P., Magistretti P.J., Barros L.F., Weber B. (2016). In vivo evidence for a lactate gradient from astrocytes to neurons. Cell Metabol..

[bib25] Naylor E., Aillon D.V., Barrett B.S., Wilson G.S., Johnson D.A., Johnson D.A., Harmon H.P., Gabbert S., Petillo P.A. (2012). Lactate as a biomarker for sleep. Sleep.

[bib26] Papatheodoropoulos C., Sotiriou E., Kotzadimitriou D., Drimala P. (2007). At clinically relevant concentrations the anaesthetic/amnesic thiopental but not the anticonvulsant phenobarbital interferes with hippocampal sharp wave-ripple complexes. BMC Neurosci..

[bib27] Pellerin L., Pellegri G., Bittar P.G., Charnay Y., Bouras C., Martin J.L., Stella N., Magistretti P.J. (1998). Evidence supporting the existence of an activity-dependent astrocyte-neuron lactate shuttle. Dev. Neurosci..

[bib28] Perez-Escuredo J., Van Hee V.F., Sboarina M., Falces J., Payen V.L., Pellerin L., Sonveaux P. (2016). Monocarboxylate transporters in the brain and in cancer. Biochim. Biophys. Acta.

[bib29] Perouansky M., MacIver M.B., Pearce R.A. (2019). Wake up, neurons! Astrocytes calling. Anesthesiology.

[bib30] Pryor K.O., Veselis R.A., Reinsel R.A., Feshchenko V.A. (2004). Enhanced visual memory effect for negative versus positive emotional content is potentiated at sub-anaesthetic concentrations of thiopental. Br. J. Anaesth..

[bib31] Ramadasan-Nair R., Hui J., Itsara L.S., Morgan P.G., Sedensky M.M. (2019). Mitochondrial function in astrocytes is essential for normal emergence from anesthesia in mice. Anesthesiology.

[bib32] Sheikhbahaei S., Turovsky E.A., Hosford P.S., Hadjihambi A., Theparambil S.M., Liu B., Marina N., Teschemacher A.G., Kasparov S., Smith J.C., Gourine A.V. (2018). Astrocytes modulate brainstem respiratory rhythm-generating circuits and determine exercise capacity. Nat. Commun..

[bib33] Shram N., Netchiporouk L., Cespuglio R. (2002). Lactate in the brain of the freely moving rat: voltammetric monitoring of the changes related to the sleep-wake states. Eur. J. Neurosci..

[bib34] Sonnay S., Duarte J.M.N., Just N., Gruetter R. (2017). Energy metabolism in the rat cortex under thiopental anaesthesia measured in Vivo by (13) C MRS. J. Neurosci. Res..

[bib35] Sonner J.M., Antognini J.F., Dutton R.C., Flood P., Gray A.T., Harris R.A., Homanics G.E., Kendig J., Orser B., Raines D.E., Rampil I.J., Trudell J., Vissel B., Eger E.I. (2003). Inhaled anesthetics and immobility: mechanisms, mysteries, and minimum alveolar anesthetic concentration. Anesth. Analg..

[bib36] Sotelo-Hitschfeld T., Niemeyer M.I., Machler P., Ruminot I., Lerchundi R., Wyss M.T., Stobart J., Fernandez-Moncada I., Valdebenito R., Garrido-Gerter P., Contreras-Baeza Y., Schneider B.L., Aebischer P., Lengacher S., San Martin A., Le Douce J., Bonvento G., Magistretti P.J., Sepulveda F.V., Weber B., Barros L.F. (2015). Channel-mediated lactate release by K(+)-stimulated astrocytes. J. Neurosci..

[bib37] Theparambil S.M., Ruminot I., Schneider H.P., Shull G.E., Deitmer J.W. (2014). The electrogenic sodium bicarbonate cotransporter NBCe1 is a high-affinity bicarbonate carrier in cortical astrocytes. J. Neurosci..

[bib38] Thrane A.S., Rangroo Thrane V., Zeppenfeld D., Lou N., Xu Q., Nagelhus E.A., Nedergaard M. (2012). General anesthesia selectively disrupts astrocyte calcium signaling in the awake mouse cortex. Proc. Natl. Acad. Sci. U. S. A..

[bib39] Turovsky E., Theparambil S.M., Kasymov V., Deitmer J.W., Del Arroyo A.G., Ackland G.L., Corneveaux J.J., Allen A.N., Huentelman M.J., Kasparov S., Marina N., Gourine A.V. (2016). Mechanisms of CO2/H+ sensitivity of astrocytes. J. Neurosci..

[bib40] Vacas S., Kurien P., Maze M. (2013). Sleep and anesthesia - common mechanisms of action. Sleep Med Clin.

[bib41] Veselis R.A., Feshchenko V.A., Reinsel R.A., Dnistrian A.M., Beattie B., Akhurst T.J. (2004). Thiopental and propofol affect different regions of the brain at similar pharmacologic effects. Anesth. Analg..

[bib42] Voss L.J., Harvey M.G., Sleigh J.W. (2016). Inhibition of astrocyte metabolism is not the primary mechanism for anaesthetic hypnosis. SpringerPlus.

[bib43] Watson C.J., Baghdoyan H.A., Lydic R. (2010). Neuropharmacology of sleep and wakefulness. Sleep Med Clin.

[bib44] Wechsler R.L., Dripps R.D., Kety S.S. (1951). Blood flow and oxygen consumption of the human brain during anesthesia produced by thiopental. Anesthesiology.

[bib45] Wentlandt K., Samoilova M., Carlen P.L., El Beheiry H. (2006). General anesthetics inhibit gap junction communication in cultured organotypic hippocampal slices. Anesth. Analg..

